# RNA-seq approach to analysis of gene expression profiles in dark green islands and light green tissues of *Cucumber mosaic virus*-infected *Nicotiana tabacum*

**DOI:** 10.1371/journal.pone.0175391

**Published:** 2017-05-10

**Authors:** Lijuan Chen, Chunyan Fei, Lin Zhu, Zhenpeng Xu, Wenshan Zou, Ting Yang, Honghui Lin, Dehui Xi

**Affiliations:** Ministry of Education Key Laboratory for Bio-Resource and Eco-Environment, College of Life Science, Sichuan University, Chengdu, China; National University of Singapore, SINGAPORE

## Abstract

Dark green islands (DGIs) surrounded by light green tissues (LGTs) are common leaf symptoms of plants that are systemically infected by various viruses that induce leaf mosaic in infected plants. The inoculation of *Cucumber mosaic virus* (CMV) in *Nicotiana tabacum* produced a commonly occurring sequence of classic patterns of DGIs and LGTs. Previous studies confirmed that there are significant differences between DGIs and LGTs in terms of physiology, biochemistry and molecular biology, but the mechanisms by which DGIs form remain unclear. To investigate the global gene expression changes that occur in these special tissues, individual differential gene expression tag libraries were constructed from three total RNA samples isolated from DGIs, LGTs and control plants (CK) and were sequenced using an Illumina HiSeq^TM^ 2000. An analysis of differentially expressed genes (DEGs) and Gene Ontology (GO) and Kyoto Encyclopedia of Genes and Genomes (KEGG) pathway enrichment analysis were performed. These analyses revealed the differences between DGIs, LGTs and CK. GO enrichment and KEGG pathway analyses suggested that several pathways related to photosynthesis and chlorophyll metabolism were enriched in DGIs compared to LGTs and CK. Several pathways related to apoptosis were significantly up-regulated in LGTs compared to DGIs. Additionally, we identified sets of DEGs that may be related to the formation or development of DGIs and LGTs. Our systematic analyses provide comprehensive transcriptomic information regarding DGIs and LGTs in CMV-infected *N*. *tabacum*. These data will help characterize the detailed mechanisms of DGI and LGT formation.

## Introduction

Viruses can cause a variety of diseases in plants, and virus infection results in a range of symptoms. Mosaic viruses are plant viruses that induce leaf mosaic in infected plants; this syndrome includes the formation of discrete regions of dark green tissues [1]. The dark green tissues are also called dark green islands (DGIs), which are surrounded by yellow or light green tissues (LGTs) [1–3]. DGIs, which are somewhat of a mystery in plants, have attracted the attention of many researchers [4]. Therefore, extensive literature is available concerning the comparison of DGIs and LGTs [5–7].

Previous studies indicated that DGIs typically possess more than one cell layer and can also contain multiple cells that they were produced by a single cell division [5]. The cells within the DGIs have been demonstrated to be free of viral RNAs and proteins. However, those cells are connected to infected cells in LGTs through plasmodesmata [5]. The formation of DGIs on leaves is complex and can occur randomly in any direction [8, 9]. Moreover, there is no definite causal agent for DGI formation, and no specific method defines the final size of DGIs [9]. DGIs can vary in size and position on systemically infected leaves [9]. Additionally, several factors, including the virus strain, the leaf age, the season of the year, and the time of infection, can influence the number of DGIs on systematic leaves [9]. Moreover, DGIs were found to be resistant to reinfection by the same or other strains of challenging viruses that induce mosaic symptoms but were susceptible to infection by dissimilar viruses [3, 10, 11]. Several studies also revealed that DGI cells exhibit cytology similar to that of healthy leaf cells [12–14]. In contrast, the cells within LGTs are usually deformed [5, 12, 15]. For instance, LGT cells are smaller than cells in healthy tissues or DGIs, and the arrangement of LGT cells is often irregular and disordered [12]. It has been shown that recovered tissues undergo posttranscriptional degradation of viral and transgene RNA [16]. Moore et al. (2001) proposed that similar to recovered tissues, a post-transcriptional gene silencing (PTGS) mechanism could be responsible for the maintenance of DGIs [14]. Our previous study also suggested that there are significant differences between DGIs and LGTs in terms of physiology, biochemistry and molecular biology [13]. Additionally, DGIs were more similar to recovered tissues and could resist viral infection better than LGTs, which is a prediction that is supported by most DGI characteristics [13].

*Cucumber mosaic virus* (CMV), which is a type of mosaic virus, has a worldwide distribution and a very wide host range [17]. The symptoms of CMV can vary greatly depending on the species and age of the plants [18]. The interaction between *Nicotiana tabacum* and CMV provides a commonly occurring sequence of classic patterns of DGIs and LGTs as well as special tissues in successive expanded leaves at 6–10 weeks post-inoculation. In recent years, the development of RNA-sequencing (RNA-seq) technology has provided a rapid and cost-effective platform for measuring differences in gene expression [19, 20]. This new technology revolutionized the procedures that are used to analyze gene expression levels [20–22], which are important for evaluating specific, different, and abundant genes from millions of sequencing tags in experimental models.

Digital gene expression (DGE) exploration of the special tissues formed on CMV-infected *N*. *tabacum* plants have rarely been reported. In the present study, an RNA-seq method was used in combination with DGE profile analysis to investigate the gene expression changes that occur in DGIs and LGTs. Our preliminary results provided new insights at the molecular and cellular levels and identified the biological events associated with the differences between DGIs and LGTs. This evidence may broaden our understanding of the mechanisms by which these tissues form.

## Materials and methods

### Plant cultivation and virus inoculation

*N*. *tabacum cv*. *NC89* plants were grown in a temperature-controlled growth chamber at an irradiation dose of 60 μM m-^2^ s-^1^ on a 12-h light/12-h dark cycle with an average temperature of 23°C. After approximately 5 weeks of growth, two leaves from the bottom insertions were mechanically inoculated with CMV-AH, as described previously [23]. Corresponding leaves from the control plants were mock-inoculated with phosphate buffer. Overall, 160 and 100 tobacco plants were CMV-inoculated and mock-inoculated, respectively. Sample leaves were obtained from the mock-inoculated and CMV-infected plants at 20 days post-inoculation (dpi), and at least 80 DGIs and 80 LGTs regions from different plants were cut out with a sterile scalpel, frozen immediately in liquid nitrogen and stored at -80°C until RNA extraction for DGE sequencing.

### RNA sequencing and transcriptome data analysis

Illumina sequencing was carried out at Novogene Bioinformatics Technology Co., Ltd., in Beijing, China. Equal quantities of total RNA from the three samples (DGIs, LGTs and CK) were mixed to prepare the pooled RNA sample for RNA-Seq. Total RNA was isolated using the TRIzol reagent (Invitrogen, Carlsbad, CA, USA) according to the manufacturer’s recommendations. Then, RNA degradation and contamination were monitored on 1% agarose gels. We evaluated the RNA purity using a NanoPhotometer® spectrophotometer (IMPLEN, Westlake Village, CA, USA). Moreover, the Qubit® RNA Assay Kit was used in a Qubit® 2.0 Fluorometer (Life Technologies, CA, USA) to measure the RNA concentration. RNA integrity was assessed with the RNA Nano 6000 Assay Kit of the Agilent Bioanalyzer 2100 system (Agilent Technologies, CA, USA).

Sequencing libraries were generated using the NEBNext® Ultra™ RNA Library Prep Kit for Illumina® (NEB, USA), following the manufacturer’s protocol. Index codes were added to attribute sequences to each sample. First, mRNA was purified from the total RNA with poly-T oligo-attached magnetic beads. Fragmentation was carried out using divalent cations under an elevated temperature in NEBNext First Strand Synthesis Reaction Buffer (5X). Next, a random hexamer primer and M-MuLV Reverse Transcriptase (RNase H^-^) were used to synthesize the first strand cDNA. Then, second strand cDNA synthesis was performed using DNA Polymerase I and RNase H. Exonuclease/polymerase activities were used to convert the remaining overhangs into blunt ends. After adenylation of the 3’ ends of the DNA fragments, the NEBNext Adaptor with hairpin loop structures was ligated to prepare for hybridization. To select cDNA fragments of 150–200 bp in length, the AMPure XP system (Beckman Coulter, Beverly, USA) was used. Next, 3 μl of the USER Enzyme (NEB, USA) was used with size-selected, adaptor-ligated cDNA at 37°C for 15 min, followed by a 5 min incubation at 95°C before PCR. PCR was then performed with the Phusion High-Fidelity DNA polymerase, Universal PCR primers and the Index (X) Primer. Finally, the PCR products were purified (AMPure XP System), and the library quality was assessed using the Agilent Bioanalyzer 2100 system.

Clustering of the index-coded samples was performed using the cBot Cluster Generation System and the TruSeq PE Cluster Kit v3-cBot-HS (Illumina), according to the manufacturer’s instructions. After cluster generation, the library preparations were sequenced on an Illumina Hiseq 2000 platform, and 125 bp paired-end reads were generated.

Raw data (raw reads) in fastq format were first processed through in-house Perl scripts. Clean data (clean reads) were obtained by removing reads containing adapter sequences, reads containing poly-N, and low-quality reads from raw data. The Q20, Q30, GC-content, and sequence duplication level of the clean data were calculated. All downstream analyses were based on high-quality clean data.

The left files (read1 files) from all libraries/samples were pooled into one large left.fq file. The right files (read2 files) were pooled into one large right.fq file. Transcriptome assembly was accomplished based on the left.fq and right.fq files using program Trinity with min_kmer_cov set to 2 by default and all other parameters set to the default values [24]. Gene functions were annotated based on the following databases: the non-redundant (NR, NCBI) protein database, the nucleotide sequence (NT, NCBI) database, the Protein family (PFAM), the Swiss-Prot database, the Kyoto Encyclopedia of Genes and Genomes Ortholog (KO) database, the Clusters of euKaryotic Ortholog Groups of proteins (KOG, NCBI) database and the Gene Ontology (GO) database.

### Library preparation for DGE sequencing and DGE data analysis

Total RNA was isolated from the three samples (DGIs, LGTs and CK) stored at -80°C using the TRIzol reagent (Invitrogen, Carlsbad, CA, USA). The methods used for RNA extraction, RNA quantification, RNA qualification, library preparation and clustering for DGE sequencing were the same as those described above for transcriptome sequencing. The library preparations were also sequenced on an Illumina HiSeq^TM^ 2000 platform, and 50 bp single-end reads were generated. In addition, the quality control procedures were similar to the quality control procedures described above for transcriptome sequencing.

Gene expression levels were estimated via RSEM [25] for each sample. The bowtie parameter mismatch was 2. Clean data were mapped back onto the assembled transcriptome, and a read count for each gene was obtained from the mapping results.

In RNA-seq, FPKM (expected number of Fragments Per Kilobase of transcript sequence per Millions base pairs sequenced), considers the effects of sequencing depth and gene length for the reads count simultaneously. In currently, it is the most common method of for estimating levels of gene expression [26]. Therefore, we made a FPKM conversion for the read count.

Because there were no biological replicates, the read counts were adjusted for each sequenced library using the edgeR program package through one scaling normalized factor prior to differential gene expression analysis [27–29]. Differential expression analysis of two samples was performed using the DEGseq (2010) R package. The P-value was adjusted using the q-value [30]. Corrected P-values were also called q-values. A q-value<0.005 and log2(fold_change)|>1 were set as the thresholds for significant differential expression [31].

GO enrichment analysis of the differentially expressed genes (DEGs) was implemented using the GOseq R packages based on the Wallenius non-central hyper-geometric distribution [32, 33], which can adjust for gene length bias in DEGs.

The Kyoto Encyclopedia of Genes and Genomes (KEGG), which is a valuable database resource, was used to facilitate our understanding of the high-level functions and utilities of various levels of the biological system, including the cell, organism and ecosystem levels, from molecular-level information, especially large-scale molecular datasets generated by genome sequencing and other high-throughput experimental technologies (http://www.genome.jp/kegg/) [34]. The KOBAS (2.0) software was used to detect the statistical enrichment of DEGs in KEGG pathways [35]. In KEGG pathway analysis, the formula for calculating enriched P-values was:

$$\text{P}=1-\sum_{i=0}^{m-1}\frac{\left(\begin{array}{c} M\\ i \end{array}\right)\left(\begin{array}{c} N-M\\ n-i \end{array}\right)}{\left(\begin{array}{c} N\\ n \end{array}\right)}$$

Here, N is the total number of genes that had KEGG annotations, n is the number of DEGs in N, M is the total number of genes annotated to specific pathways, and m is number of DEGs in M. A P-value of 0.05 was selected as the threshold for deciding whether a gene set was significantly enriched.

### Leaf-press blotting and paraffin sections

For the leaf-press blotting assays, systemic CMV-infected *N*. *tabacum* leaves with typical DGIs and LGTs were pressed onto nitrocellulose membranes, using the method described by Gal-On et al (1994), with some modifications [36]. The subsequent steps were the same as the steps used for protein blotting [37]. Paraffin sections were collected as described previously, with several modifications [38].

### Measurement of chlorophyll fluorescence parameters and chlorophyll content

The content of chlorophylls (Chl) a and b was determined according to the methods described by Kichtenthaler and Wellburn [39]. The Chl fluorescence parameters of the leaves were measured using an FMS2 fluorescence meter (PAM-2100, Walz, Germany) according to previously described methods [13].

### Determination of electrolyte leakage, methane dicarboxylic aldehyde and hydrogen peroxide

Electrolyte leakage and methane dicarboxylic aldehyde (MDA) content viassayed as described previously [37]. Endogenous concentrations of hydrogen peroxide (H_2_O_2_) were detected as described previously [40]. H_2_O_2_ concentrations were determined using the Amplex red hydrogen peroxide/peroxidase assay kit (Invitrogen).

### Determination of cytokinin, abscisic acid and indole acetic acid

The endogenous level of cytokinin (CTK) was quantified via high-performance liquid chromatography-mass spectrometry (HPLC-MS), according to previously described methods [13]. The compound 6-benzylaminopurine (6-BA) was obtained from Sigma-Aldrich and used as an internal standard. Abscisic acid (ABA) and indole acetic acid (IAA) concentrations were measured via an enzyme-linked immunosorbent assay (ELISA), using the protocols described by Teng et al. (2006) and Chen et al. (2009), respectively [41, 42].

### Quantitative real-time polymerase chain reaction validation

Validation of the RNA-seq data for 12 different genes was performed using quantitative real-time polymerase chain reaction (qRT-PCR) analysis (Bio-Rad Laboratories, Hercules, CA, USA). The qRT-PCR analysis was performed using 3 RNA samples: the RNA sample used in the DGE experiment and two other replicates from new plant materials. Primers for selected genes were designed using the Primer-Premier 5 software (PREMIER Biosoft, Palo Alto, CA, USA). The primer pairs are summarized in S1 Table. The cDNA was amplified using SYBR Premix Ex Taq (TaKaRa). The amplification of the target genes was monitored every cycle based on SYBRgreen I fluorescence. The relative quantification of the target gene expression level was achieved using the comparative *Ct* method [43]. Three technical replicates were performed for each experiment. The amplification of the elongation factor 1a gene (*EF1a*) was used as an internal control.

### Statistical analyses

The results are expressed as the means of at least three independent measurements, and the average results are presented with standard deviations (n = 3). The student’s *t*-test was used for comparisons between different samples. Differences were considered to be statistically significant when *P <*0.05.

## Results

### Annotation of unigenes in *N*. *tabacum*

**Fig 1:**
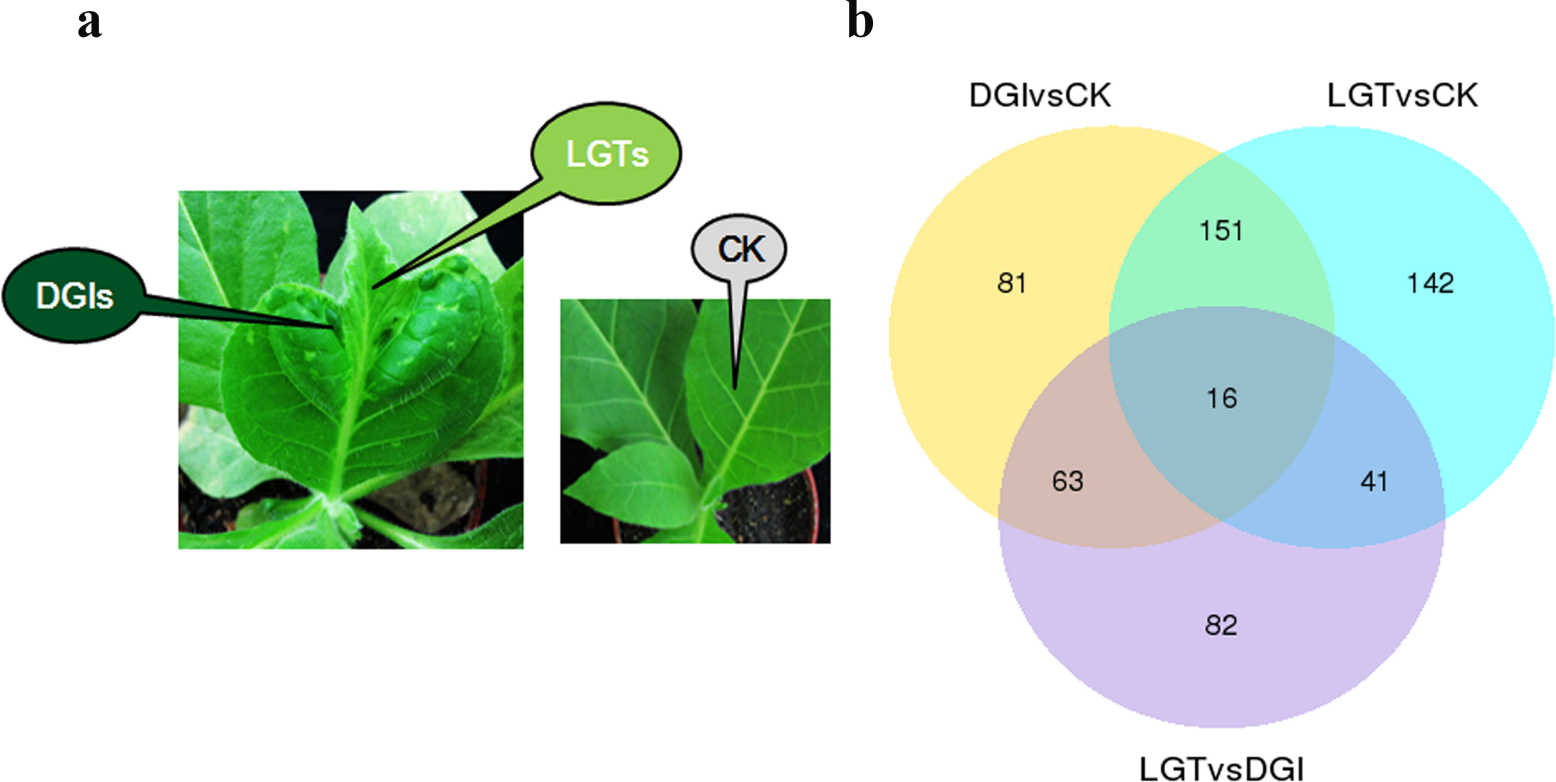
**(a) Symptoms of *N*. *tabacum* leaves systemically infected with CMV**. CK, *N*. *tabacum* were mock inoculated with phosphate buffer; DGI, dark green tissues of CMV-infected *N*. *tabacum*; LGT, light green tissue of CMV- infected *N*. *tabacum*; (**b**) **Venn diagram of DEGs between three comparisons (DGI vs. CK, LGT vs. CK and LGT vs. DGI).** The sum of the numbers in each large circle represents total number of differentially expressed genes between combinations, the overlap part of the circles represents common differentially expressed genes between combinations.

When *N*. *tabacum* was systemically infected with CMV, DGIs formed and gave rise to symptoms that included chlorosis of the leaves surrounding discrete regions of DGIs (Fig 1A). The appearance of DGIs and LGTs differed significantly. Several previous studies indicated that significant differences exist between DGIs and LGTs [2, 4, 6, 10, 13, 44]. One of the most significant differences between these types of tissues is virus accumulation. Leaf-press blotting showed directly that virus accumulation in LGTs was much higher than virus accumulation in DGIs (S1 Fig). To thoroughly analyze the differences between these special tissues, RNA-Seq was used to profile transcriptional changes.

Since the genome sequence of *N*. *tabacum* is not available, we first defined the transcriptome of *N*. *tabacum* using RNA-Seq. Equal quantities of total RNA from the three samples (DGIs, LGTs and CK) were pooled and used for library construction. The library was sequenced on an Illumina HiSeq^TM^ 2000 platform. A total of 107,359, 006 clean reads (from a total of 110,411,642 raw reads) were generated using Illumina paired-end sequencing (S1 Data) and assembled into 156,074 transcripts. The 156,074 transcripts were assembled according to average and N50 lengths of 892 and 1585 bp, respectively (S2 Fig). After gap filling with paired-end reads, the transcripts were assembled into 106,429 unigenes. The unigene length distribution is displayed in S3 and S4 Figs. The N50 length of the unigenes was 1116 bp, and the N90 length was 269 bp. The BLAST results for the unigenes were compared against seven databases, including NR, NT, PFAM, the Swiss-Prot database, the KO database, the KOG database and the GO database. The results are shown in S2 Data.

The gene functions of the sequences with BLAST hits were annotated based on three databases: KOG, KO and KEGG pathway. KOG analysis indicated that 12,151 unigenes had KOG annotations and could be grouped into 26 clusters (S5 Fig). In contrast, 27,618 unigenes had GO annotations and were distributed among 48 functional classes (S6 Fig). 10132 unigenes with KEGG annotations were distributed in 272 KEGG pathways. Signal transduction, carbohydrate metabolism and translation were the dominant KEGG pathways (S7 Fig).

### Identification of DEGs in DGIs, LGTs and CK

To better understand these special tissues, DEGs of transcripts in *N*. *tabacum* leaves after mock inoculation (CK) and CMV inoculation (DGIs and LGTs) were examined using an Illumina platform. A total of 13,810,892 (CK), 12,791,386 (DGIs), 12,910,775 (LGTs) sequence reads were generated. A sequencing summary is provided in Table 1 and S8 Fig.

**Table 1:** Statistics of DGE sequencing.

Sample	Raw Reads	Clean Reads	Clean Bases	Error(%)	Q20(%)	Q30(%)	GC Content(%)
CK	13810892	13748924	0.69G	0.01	99.03	96.81	42.36
DGI	12791386	12739290	0.64G	0.01	99.02	96.78	42.49
LGT	12910775	12855148	0.64G	0.01	99	96.74	42.37

The transcriptome sequences assembled by Trinity were used as reference sequences [45, 46]. The clean data were mapped back onto the assembled transcriptome using the RSEM software [25]. The bowtie parameter mismatch was 2. In the CK, DGIs and LGTs, 92.57%, 92.14%, and 92.22% of the total reads from DGE were mapped to the reference sequences, respectively (Table 2).

**Table 2:** DGE reads mapped to the reference sequences.

Sample name	Total reads	Total mapped
CK	13748924	12727858 (92.57%)
DGI	12739290	11738400 (92.14%)
LGT	12855148	11855547 (92.22%)

The normalized expression levels of control and *CMV*-infected plants were compared to detect differentially expressed genes. A Venn diagram showed the distribution of DEGs among the three comparisons (Fig 1B). A total of 311 DEGs were obtained after a comparison of DGIs to CK (DGIs vs. CK). Overall, 350 DEGs were identified after comparing LGTs to CK (LGTs vs. CK). In comparison, only 202 DEGs were identified after a comparison between LGTs and DGIs (LGTs vs. DGIs). In total, 81, 142 and 82 DEGs were specific for DGIs vs. CK, LGTs vs. CK and LGTs vs. DGIs, respectively. Curiously, the LGTs vs. CK comparison showed a greater number of total and specific differentially expressed genes in comparison to the DGIs vs. CK comparison, including the genes that encode cytochrome P450 (c47617_g1, c51201_g3, c44961_g1), probable *WRKY* transcription factor 40 (c46829_g1, c51556_g2, c40144_g1, c40144_g3) and probable xyloglucan endotransglucosylase (c47038_g4, c36196_g1, c19467_g2). In addition, in DGIs, 295 (95%) and 16 (5%) of the 311 genes were up-regulated and down-regulated, respectively, relative to CK (S3 and S4 Data). In LGTs, 301 (86%) and 49 (14%) of the 350 genes were up-regulated and down-regulated, respectively, relative to CK (S5 and S6 Data). However, only 201 DEGs were detected in the LGTs vs. CK comparison; of those DEGs, 39 (19%) were up-regulated and 163 (81%) were down-regulated (S7 and S8 Data).

### Functional annotation of DEGs

**Fig 2:**
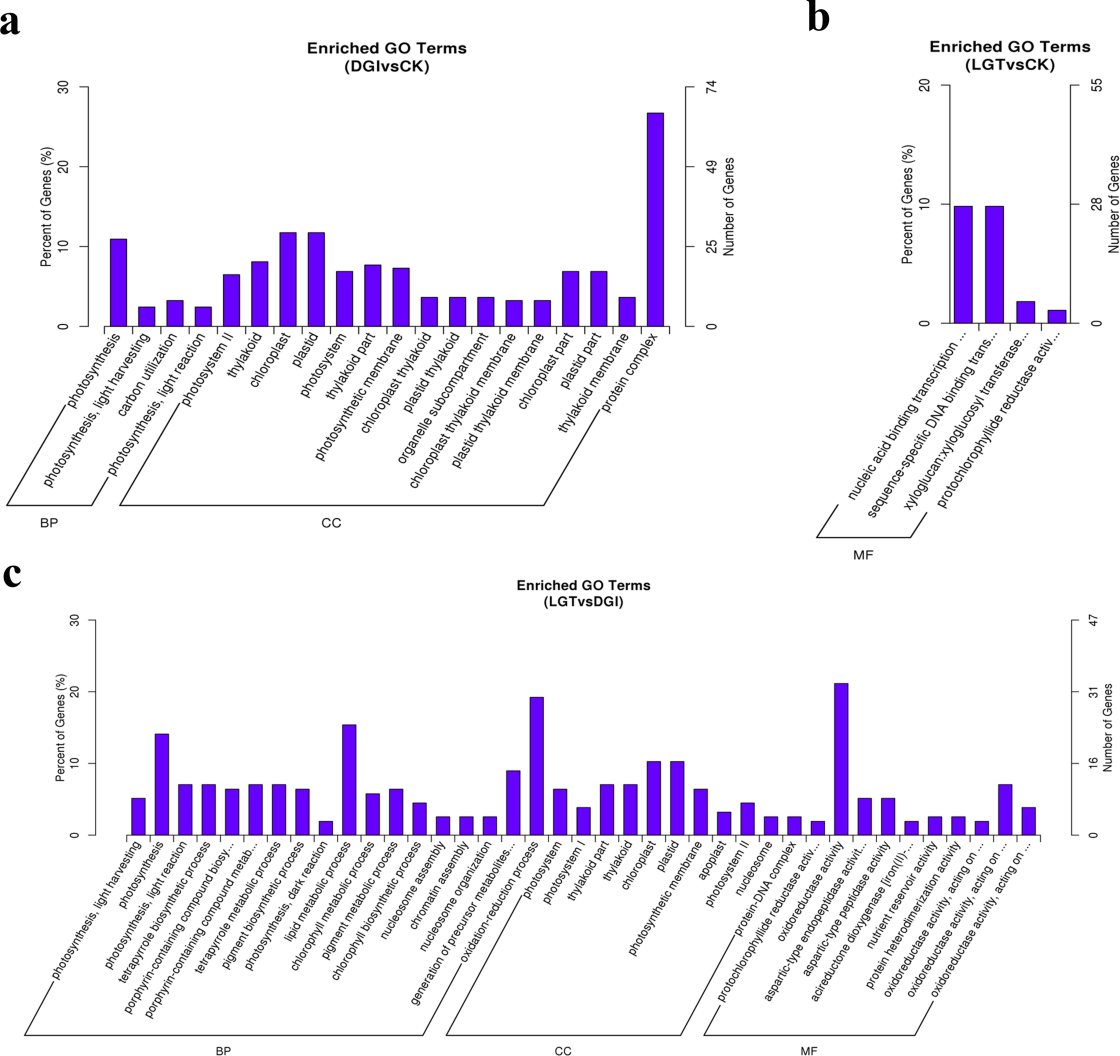
Histogram of Gene Ontology classification. The results are summarized in three main categories: biological process (BP), cellular component (CC), and molecular function (MF). The left Y-axis indicates the percentage of a specific category of genes in that main category. The right Y-axis indicates the number of genes in a category.

To obtain a functional categorization of the DEGs, GO analysis was used to classify the DEGs. Of the 311 total DEGs identified in the DGIs vs. CK analysis and the 350 total DEGs identified in the LGTs vs. CK analysis, 247 and 275 DEGs, respectively, were assigned one or more GO terms (S9 and S10 Data). Twenty significantly different GO annotations were obtained from the DGIs vs. CK analysis (Fig 2A), with four annotations belonging to biological processes and sixteen annotations belonging to cellular components. However, a GO analysis of these genes did not reveal significantly enriched groups of genes belonging to the molecular function categories. With 27 genes, photosynthesis (GO: 0015979) was dominant in the main category of biological processes. A protein complex (GO: 0043234) that consisted of 66 genes was dominant in the main category of cellular components. We also observed that a high percentage of genes from functional groups for chloroplasts (GO: 0009507), with 29 genes, and plastids (GO: 0009536), with 29 genes, were enriched. In contrast, only four significantly different GO annotations belonging to the molecular function categories were enriched in LGTs vs. CK (Fig 2B). Nucleic acid binding transcription factor activity (GO: 0001071), with 27 genes, and sequence-specific DNA binding transcription factor activity (GO: 0003700), with 27 genes, were dominant in the molecular functions category.

The 156 selected significant DEGs in the LGTs vs. DGIs comparison were categorized into 39 functional groups that were clustered in three main GO classification categories (biological processes, cellular components, and molecular functions) (Fig 2C, S11 Data). Biological processes made up the majority of the GO annotations (18 significantly enriched groups), followed by cellular components (11 significantly enriched groups) and molecular functions (10 significantly enriched groups). Among 18 different biological process categories, the oxidation-reduction process (GO: 0055114), with 30 genes, the lipid metabolic process (GO: 0006629), with 24 genes, and photosynthesis (GO: 0015979), with 22 genes, were the three most frequently identified GO terms for the genes that were part of the biological processes component. Within the cellular components, the major categories were chloroplasts (GO: 0009507) (16 genes) and plastids (GO: 0009536) (16 genes). Additionally, oxidoreductase activity (GO: 0016491) (33 genes) was dominant in the main category of molecular functions.

**Fig 3:**
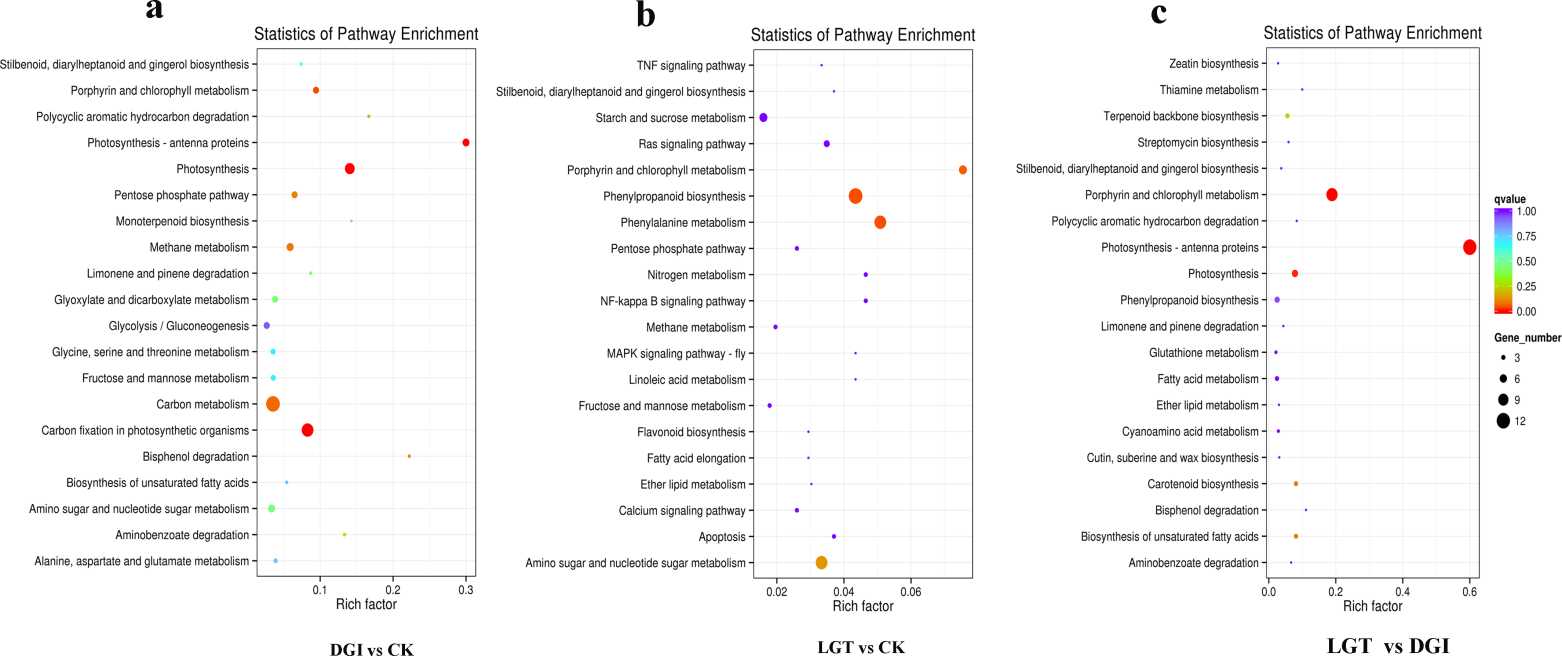
Functional classification and pathway assignment of unigenes by KEGG.

In addition to GO assignments, KEGG pathway mapping based on KO terms for assignments was also carried out with the total list of DEGs from the three comparisons. A total of 311 DEGs in the DGIs vs. CK comparison were assigned to 58 KEGG pathways. The top 20 enriched pathways were displayed in Fig 3A. The results indicated that the most frequently represented pathways were ‘photosynthesis-antenna proteins’ (PATH: ko00196), ‘photosynthesis’ (PATH: ko00195), ‘carbon fixation in photosynthetic organisms’ (PATH: ko00710), and ‘porphyrin and chlorophyll metabolism’ (PATH: ko00860), with 6, 9, 11 and 5 members, respectively.

The top 20 enriched pathways for LGTs vs. CK and LGTs vs. DGIs are displayed in Fig 3B and 3C, respectively. The pathways with the greatest representation in LGTs vs. CK were the phenylpropanoid biosynthesis pathway (PATH: ko00940), with 7 members, and phenylalanine metabolism (PATH: ko00360), with 6 members. The most frequently represented pathways in LGTs vs. DGIs were photosynthesis-antenna proteins (PATH: ko00196), porphyrin and chlorophyll metabolism (PATH: ko00860), and photosynthesis (PATH: ko00195), with 12, 10 and 5 members, respectively.

### Validation of DGE data by qRT-PCR

**Fig 4:**
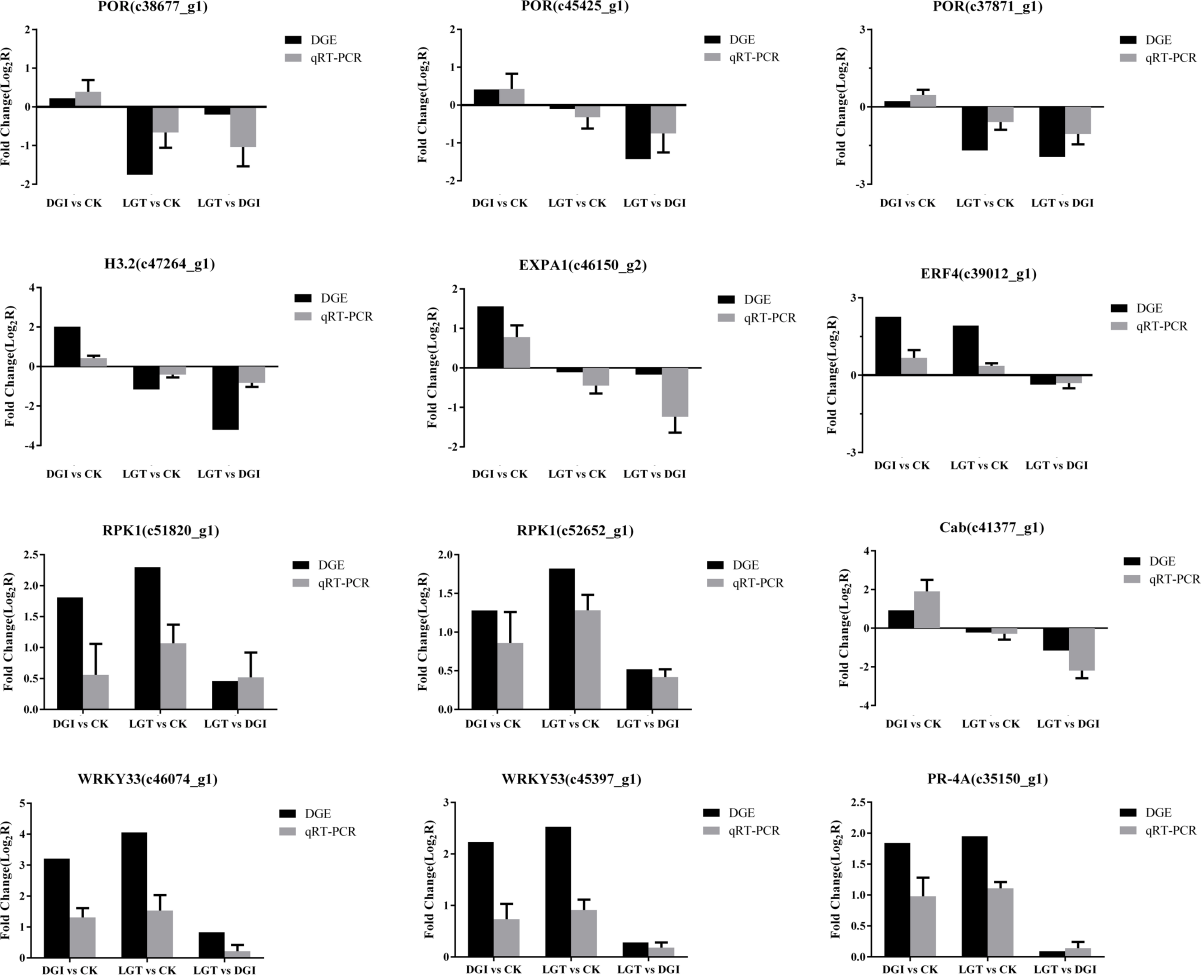
Validation of the DGE analysis by quantitative real-time polymerase chain reaction (qRT-PCR). Error bars represent the standard deviations of qRT-PCR signals (n = 3).

Validation of the DGE data for 12 unigenes with annotations was performed via qRT-PCR (Fig 4). The results showed that the qRT-PCR data for these genes were consistent with the DGE results. For example, both qRT-PCR and DGE analysis showed that the genes encoding expansin-A1 (EXPA1 c46150_g2), protochlorophyllide reductase (POR c45425_g1, 38677_g1, 37871_g1) and histone (H3.2 c47264_g1) were highly expressed in DGIs, while these genes were significantly suppressed in LGTs compared to CK. Additionally, the DGE results indicated that the genes encoding probable LRR receptor-like serine/threonine-protein kinase (RPK1 c51820_g1, c52652_g1), probable WRKY transcription factors (WRKY33 c46074_g1, WRKY53 c45397_g1) and ethylene-responsive transcription factor 4 (ERF4 c39012_g1) were significantly up-regulated in DGIs and LGTs compared to CK, which was consistent with the qRT-PCR expression patterns (Fig 4). Moreover, the expression levels of the genes encoding pathogenesis-related protein (PR-4A c35150_g1) and chlorophyll a/b binding protein (Cab c41377_g1) that were revealed by DGE analysis were also verified by the qRT-PCR analysis (Fig 4). Thus, the results of the qRT-PCR analysis were very similar to those obtained via RNA-seq analysis, which indicated that the changes in expression detected by RNA-seq were accurate.

### Expression of photosynthesis- and photosystem-related genes was up-regulated in DGIs

In the present study, we identified a substantial number of genes involved in different steps of photosynthesis and the photosystem that were up-regulated in DGIs (Table 3). In contrast, few expression differences for these genes were observed in the LGTs vs. CK comparison. In addition, an examination of GO terms suggested that the three main categories were devoted primarily to the control of photosynthesis-related processes and chloroplast components. For example, the GO terms photosynthesis (GO: 0015979), photosynthesis, light harvesting (GO: 0009765), photosystem I (GO: 0009522), photosystem II (GO: 0009523), chloroplast (GO: 0009507), photosynthetic membrane (GO: 0034357), chloroplast thylakoid lumen (GO: 0009543) and chloroplast thylakoid (GO: 0009534) were enriched in DGIs vs. LGTs and DGIs vs. CK. The KEGG pathway analysis also indicated that several pathways related to photosynthesis were enriched in DGIs vs. LGTs and DGIs vs. CK, including porphyrin and chlorophyll metabolism (PATH: ko00860), photosynthesis-antenna proteins (PATH: ko00196), photosynthesis (PATH: ko00195), carbon fixation in photosynthetic organisms (PATH: ko00710) and carotenoid biosynthesis (PATH: ko00906).

**Table 3:** Differentially expressed (log_2_Fold_change) of photosynthesis- and photosystem-related genes in DGI vs. CK, DGI vs. LGT and LGTs vs. CK.

Gene ID	Swissprot Description	A	B	C
c44343_g2	Chlorophyll a-b binding protein 36	1.96	1.37	0.54
c44396_g1	Chlorophyll a-b binding protein 13	1.85	1.08	0.78
c48271_g1	Chlorophyll a-b binding protein CP24	1.59	0.71	0.90
c37314_g1	Photosystem II repair protein PSB27-H1	1.48	0.71	0.79
c44343_g1	Chlorophyll a-b binding protein 37	1.42	1.37	0.07
c35705_g1	Photosystem II 5 kDa protein	1.40	1.07	0.35
c38029_g1	Thylakoid lumenal 16.5 kDa protein	1.16	1.07	0.11
c41377_g2	Chlorophyll a-b binding protein 4	1.12	1.73	-0.58
c42990_g1	Chlorophyll a-b binding protein 8	1.11	1.13	0.02
c41707_g1	Photosystem II core complex proteins psbY	1.10	0.93	0.19
c38916_g1	Photosystem I reaction center subunit II	1.09	0.89	0.21
c19662_g1	Photosystem II reaction center W protein	1.05	0.67	0.40
c44710_g2	Photosystem I reaction center subunit N	1.05	1.11	-0.04
c41377_g1	Chlorophyll a-b binding protein P4	0.92	1.16	-0.22
c42082_g1	Chlorophyll a-b binding protein CP26	0.79	1.09	-0.27
c42990_g2	Chlorophyll a-b binding protein 8	0.59	1.02	-0.42
c43018_g1	Chlorophyll a-b binding protein CP29.2	0.94	1.18	-0.22
c43253_g1	Chlorophyll a-b binding protein 6A	0.58	1.02	-0.42
c48649_g1	Chlorophyll a-b binding protein 21	0.72	1.04	-0.31
c48649_g2	Chlorophyll a-b binding protein 16	0.77	1.10	-0.31
c8676_g1	Photosystem I reaction center subunit VI	0.89	1.06	-0.15

“**A**” represents the log_2_Fold_change (DGI vs. CK), “**B**” represents the log_2_Fold_change (DGI vs. LGT) and “**C**” represents the log_2_Fold_change (LGT vs. CK).

Additionally, we also detected the chlorophyll contents in these tissues. The results suggested that the level of chlorophyll in DGIs was significantly higher than that observed in LGTs (S9A Fig). Meanwhile, the chlorophyll fluorescence parameters (Fm, Fv/Fm and Y(NO)) of the DGIs closely resembled those of CK, while these parameters were significantly impaired in LGTs (S9B Fig). Our previous study also suggested that DGIs contain perfect chloroplasts but LGTs do not [13]. Taken together, these results were consistent with the DGE analysis, which indicated that DGIs have a relatively normal photosynthetic system and photosynthesis in comparison to LGTs.

### LGTs suffered more serious damage than DGIs

Apoptosis is a process of programmed cell death that occurs in multicellular organisms. Apoptosis is the outcome of severe injuries caused by changes in the environment of affected cells [47], as well as changes involved in defense, development and the stress response in plants [48]. The KEGG pathway analysis indicated that several pathways related to apoptosis, including apoptosis (PATH: ko04210), the NF-kappa B signaling pathway (PATH: ko04064) and the ras signaling pathway (PATH: ko04014), were enriched in LGTs vs. DGIs and LGTs vs. CK. In addition, the TNF signaling pathway (PHAT: ko04668), which refers to a group of cytokines that can cause apoptosis [49, 50], was also up-regulated in LGTs vs. CK. These results suggested that LGTs are more seriously damaged by virus attack than DGIs.

Paraffin sectioning showed that the histology within DGIs was very similar to that of CK, whereas the histology in the LGTs surrounding the DGIs was distinct (S10 Fig). For example, similar to CK, DGIs contained relatively large numbers of cell layers, including one or two layers of elongated palisade cells and large intercellular spaces between the elongated mesophyll cells. In contrast, LGTs had relatively few cell layers and contained abnormal palisade cells, which often failed to completely elongate and contained few plastids. The contrast between palisade and spongy mesophyll cells in LGTs was inconspicuous. In addition, the spongy mesophyll cells in LGTs were irregular and replaced by large swollen vacuoles. A previous study showed that aquaporins were enriched in zones of rapid cell division and expansion [51]. DGE analysis showed that the genes encoding aquaporins were significantly up-regulated in DGIs compared to LGTs (S2 Table), which may be correlated with the elongated palisade cells observed in DGIs but not in LGTs.

Additionally, we measured two additional physiological parameters, including MDA content (or TBARS content) and electrolyte leakage. LGTs contained significantly higher levels of MDA and electrolyte leakage in comparison to DGIs and CK (S11A and S11B Fig), which suggested that the stability and integrity of the plasma membrane was significantly reduced in LGTs in comparison to DGIs. This finding coincided with the results obtained from the paraffin section experiment. Meanwhile, the total H_2_O_2_ content and the transcriptional level of respiratory burst oxidase homolog D gene (*RbohD*) were significantly up-regulated in LGTs compared to DGIs (S11C and S11D Fig). Additionally, the expression levels of genes related to antioxidant enzymes were significantly up-regulated in DGIs compared to LGTs (displayed in S2 Table). These results indicated that LGTs suffered more serious oxidative damage than DGIs and had depressed antioxidant abilities in comparison to DGIs. DEG analysis showed that many genes that were strongly up-regulated in LGTs compared to DGIs and CK, including the CMV movement protein, CMV RNA-directed RNA polymerase 2a and CMV replication protein 1a (shown in S2 Table). This finding indicated that a large amount of virus in LGTs may cause more pressure in comparison to DGIs.

### Biosynthesis and metabolism of IAA, CTK and expansins might be involved in the formation of DGIs and LGTs

The paraffin section results showed that the cells of LGTs were smaller than the cells of CK or DGIs, and the cell arrangement in LGTs was also disordered (S10 Fig). IAA and CTK had profound effects on plant growth and development, such as aspects of cell division, cell elongation and cell differentiation [52]. An analysis of DEGs indicated the genes encoding auxin binding protein 1 (ABP19A) were up-regulated in DGIs but down-regulated in LGTs in comparison with CK (S2 Table). We also detected endogenous IAA in these tissues. The results showed that the endogenous level of IAA was significantly higher in DGIs than in CK. In contrast, the IAA content of LGTs was slightly lower than that of CK (S12A Fig). Additionally, the KEGG pathway related to zeatin (a type of plant cytokinin) biosynthesis was significantly enriched in DGIs vs. LGTs and CK vs. LGTs. The DGIs exhibited much larger amounts of CTK than CK and LGTs (S12B Fig). Therefore, we hypothesized that IAA and CTK biological processes might be involved in the morphological development of DGIs and LGTs. However, further investigations are needed to clarify the roles of IAA and CTK in these tissues.

Expansins and expansin-like cell wall-loosening proteins can increase the extensibility of the cell wall [53]. According to the DEGs analysis, the genes encoding expansin A1/A4 were up-regulated in DGIs in comparison to CK and LGTs, and were also slightly down-regulated in LGTs vs. CK (S2 Table). The qRT-PCR analysis revealed that DGIs had the highest expression level for the gene encoding expansin-A1 (*EXPA1*), followed by CK (Fig 4). The cells in LGTs were smaller and were not completely elongated in comparison to the cells in DGIs and CK. Thus, we propose that expansins may have significant effects on the development of these tissues. To correlate the expression of expansin with its function in the formation of DGIs and LGTs, the responses of *EXLA4-*defective mutants (*EXLA4* RNAi), overexpression lines (35S:*EXLA4*) and wild-type plants (*WT*) after CMV infection were examined. At approximately 20-dpi, we found that the formation of DGIs in the *EXLA4* RNAi plants was repressed or reduced in comparison to *WT*. However, *EXLA4*-overexpressing plants developed more vivid and typical DGIs than *WT* plants. In addition, *EXLA4* RNAi plants were more likely to develop LGTs (S13 Fig). The results obtained from the tobacco *EXLA4* mutants indicated that the expression level of *EXLA4* could affect the formation of DGIs and LGTs on CMV-infected *N*. *tabacum* plants.

## Discussion

In this study, we used an RNA-seq and DGE approach to investigate the gene expression changes associated with the characteristic disease tissues induced in *N*. *tabacum* by CMV. We identified sets of DEGs that may be related to the formation or development of DGIs and LGTs. GO enrichment analysis and KEGG pathway analysis suggested that several pathways related to photosynthesis and chlorophyll metabolism are enriched in DGIs compared to LGTs. However, several pathways related to apoptosis are significantly up-regulated in LGTs compared to DGIs. The results obtained using tobacco expansin mutants indicated that *NtEXPA4* has effects on DGI and LGT formation. Although the two tissues both arise through interactions between CMV and *N*. *tabacum*, they involved different metabolic and biological pathways.

In 1926, Goldstein reported that the chlorophyll contents of LGTs in *Tobacco mosaic virus* (TMV)-infected *N*. *tabacum* were significantly reduced in comparison to DGIs and healthy tissues [12]. Our previous study indicated that DGIs, which were very similar to healthy tissues, contained perfect chloroplasts and possessed a developed photosynthetic capacity, in contrast to LGTs [13]. In the present study, DGE analysis indicated that many genes in pathways related to photosynthesis were up-regulated in DGIs vs. CK and DGIs vs. LGTs but were down-regulated in LGTs vs. CK. Chloroplasts, which are important organelles in plants, are key components of photosynthesis. Photosynthesis is the basic energetic driver of plant biomass production [54]. Similar to healthy tissues, DGIs had a normal photosynthetic system and photosynthesis in comparison to LGTs. The interaction system of *N*. *tabacum* and CMV was compatible. On the one hand, DGIs allow the infected plant to survive longer. On the other hand, DGIs may be a nutrient resource for virus reproduction in the surrounding LGTs. Thus, we hypothesized that DGIs represent a win-win outcome in the battle between host plants and pathogens.

An analysis of DEGs showed that the genes encoding CMV RNA-directed RNA polymerase 2a, CMV replication protein 1a and CMV movement protein were significantly up-regulated in LGTs vs. DGIs and LGTs vs. CK. This finding was somewhat consistent with the previous suggestion that DGIs were free of viral RNAs and proteins [7, 9, 13, 16]. Previous studies reported that genes related to photosynthesis and pigment metabolism were suppressed by virus infection [55]. Therefore, a high viral concentration may also be correlated with damaged photosynthesis and pigment metabolism in LGTs.

The histology analysis performed in the present study was consistent with the previous view that DGIs more closely resemble healthy tissues and contain one or two layers of elongated palisade cells, as well as large intercellular spaces between elongated mesophyll cells [12]. In contrast, in LGTs, the palisade cells were abnormal and often failed to elongate completely, with few plastids, large swollen nuclei and few intracellular spaces [12]. Palisade cells of plants contain the largest number of chloroplasts per cell and are the primary site of photosynthesis in the leaves of plants that contain them. These cells convert the energy in light to the chemical energy of carbohydrates [56]. Palisade cells abnormalities can have unfortunate effects on the development and growth of cells in LGTs through damaged photosynthesis (S9 Fig).

DGIs are more similar to recovered tissues and are resistant to super-infection by the same virus [11, 13, 14]. Recovered tissue has been shown to undergo posttranscriptional degradation of viral and transgene RNAs [16]. Dicer-like, which is a type of double-stranded (ds) RNA-specific enzyme, plays a vital role in post-transcriptional gene silencing in plants [57]. Virus-associated molecular patterns are often generically recognized by dicerlike enzymes, which then produce virus-derived small interfering RNAs (vsiRNAs) that promote antiviral defenses through RNA silencing once they are loaded into argonaute proteins [58]. According to our DEGs analysis, the gene encoding endoribonuclease dicer homolog 2 (c52671_g1) was slightly up-regulated in DGIs in comparison to LGTs (S2 Table), which indicates that DGIs represent an inhospitable internal environment for virus replication. Therefore, high expression levels of endoribonuclease dicer homolog 2 may correlate with the relatively small amount of viruses in DGIs.

Previous studies reported that expansins are plant cell wall-remodelling proteins and are involved primarily in the pH-dependent extension of plant cell walls [59]. In the present study, DEGs analysis indicated that the expression levels of genes encoding expansins are up-regulated in DGIs in comparison to LGTs. Palisade cells failed to elongate completely in LGTs but not in DGIs. In addition, a preliminary study based on tobacco *EXPA4* mutants showed that the formation of DGIs was repressed in *EXLA4* RNAi plants. Taken together, our results suggested that tobacco *EXPA4* may modulate DGI formation in CMV-infected *N*. *tabacum*. However, the underlying mechanisms by which expansin signaling affects DGI formation have yet to be determined. Further investigations are necessary to clarify these issues. Moreover, the other two phytohormones, IAA and CTK, also had profound effects on the division, elongation and differentiation of plant cells. Thus, further investigations are needed to clarify the roles of IAA and CTK in these tissues.

Overall, the combination of transcriptome sequencing and DGE analysis based on RNA-seq technology was shown to be a powerful method for identifying candidate genes involved in the response of *N*. *tabacum* to CMV. This study was also the first transcriptome study to compare DGIs and LGTs, and the obtained results will help researchers study the differences between DGIs and LGTs and the formation of these tissues in CMV-infected *N*. *tabacum* leaves. The knowledge obtained from this study will serve as a useful genetic resource for the study of DGIs and LGTs. Additionally, the identification of specific genes and the functional verification of candidate genes, including the genes that encode expansins, IAA, CTK and the dicer protein, are also needed to further analyze the formation mechanisms of disease tissues induced by mosaic viruses and to help us understand the mechanisms of plant resistance to virus infection.

## Supporting information

S1 Data Clean data QC summary. (XLS)

S2 Data BLAST results were obtained for unigenes. (XLS)

S3 Data List of all up-regulated differentially expressed genes in DGI vs. CK. (XLS)

S4 Data List of all down-regulated differentially expressed genes in DGI vs. CK. (XLS)

S5 Data List of all up-regulated differentially expressed genes in LGT vs. CK. (XLS)

S6 Data List of all down-regulated differentially expressed genes in LGT vs. CK. (XLS)

S7 Data List of all up-regulated differentially expressed genes in LGT vs. DGI. (XLS)

S8 Data List of all down-regulated differentially expressed genes in LGT vs. DGI. (XLS)

S9 Data GO enrichment of DEGs in DGI vs. CK. (XLS)

S10 Data GO enrichment of DEGs in LGT vs. CK. (XLS)

S11 Data GO enrichment of DEGs in LGT vs. DGI. (XLS)

S1 Fig Leaf-press blotting of DGI and LGT induced in *N. tabacum* by CMV. The experiments were repeated three times with similar results.
(TIF)

S2 Fig Length distribution and characterization of assembled transcripts in *N. tabacum*. (TIF)

S3 Fig Length distribution and characterization of assembled unigenes in *N. tabacum*. (TIF)

S4 Fig Unigene and transcript length distribution in *N. tabacum*. (TIF)

S5 Fig Unigene KOG annotations. Unigenes aligned to the KOG database were classified into 26 functional classes.
(TIF)

S6 Fig Unigene GO annotations. Unigenes with GO annotations were classified into three major functional categories (biological process, cellular components, and molecular functions).
(TIF)

S7 Fig Unigene KEGG annotations. Unigenes with KEGG annotations were distributed in 272 KEGG pathways.
(TIF)

S8 Fig The quality of the raw reads. (TIF)

S9 Fig The Chlorophyll content and photosynthetic fluorescence parameters of DGI and LGT induced in *N. tabacum* by CMV. The error bars represent the standard deviations of the mean values that were obtained from three biological replicates (n = 3).
(TIF)

S10 Fig Paraffin section of DGI and LGT induced in *N. tabacum* by CMV. Scale bar = 50um. The experiments were repeated three times with similar results.
(TIF)

S11 Fig **Electrolyte leakage (a), malondialdehyde (MDA) contents (b), H**_**2**_**O**_**2**_ **content (c) and transcriptional level of respiratory burst oxidase homolog D gene (*RbohD*) (d) of DGI and LGT induced in *N*. *tabacum* by CMV.** The error bars represent the standard deviations of the mean values that were obtained from three biological replicates (n = 3).
(TIF)

S12 Fig **Accumulation of IAA (a) and CTK (b) in DGIs and LGTs induced in *N*. *tabacum* by CMV.** The error bars represent the standard deviations of the mean values that were obtained from three biological replicates (n = 3).
(TIF)

S13 Fig Symptoms of tobacco leaves systemically infected with CMV. Mock, *N*. *tabacum* were mock inoculated with phosphate buffer; *EXLA4*-defective mutants (*EXLA4* RNAi); overexpression lines (35S:*EXLA4*) and wild-type plants (*WT*).
(TIF)

S1 Table *N. tabacum* gene primers be used in this study. (DOCX)

S2 Table Differentially expressed (log2Fold_change) of important genes in DGI vs. CK, LGT vs. CK and LGT vs. DGI. (DOCX)
