# Supplementary figures and images for: RNA-seq approach to analysis of gene expression profiles in dark green islands and light green tissues of Cucumber mosaic virus-infected Nicotiana tabacum
Source: PLoS One. 2017 May 10;12(5):e0175391. doi: 10.1371/journal.pone.0175391 (PMC5425015; doi:10.1371/journal.pone.0175391)

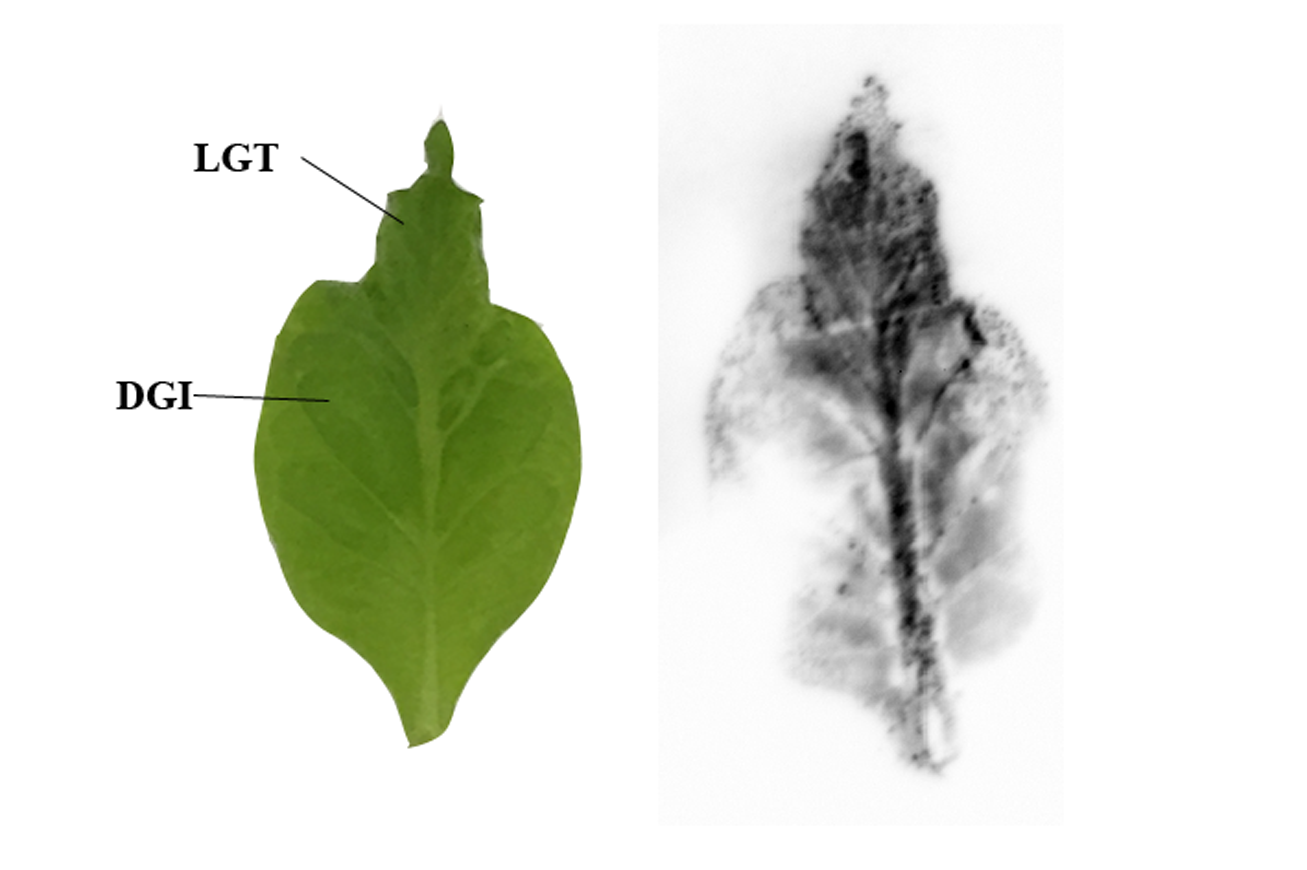

Supplement: S1 Fig — The experiments were repeated three times with similar results. (TIF) [file pone.0175391.s012.tif]

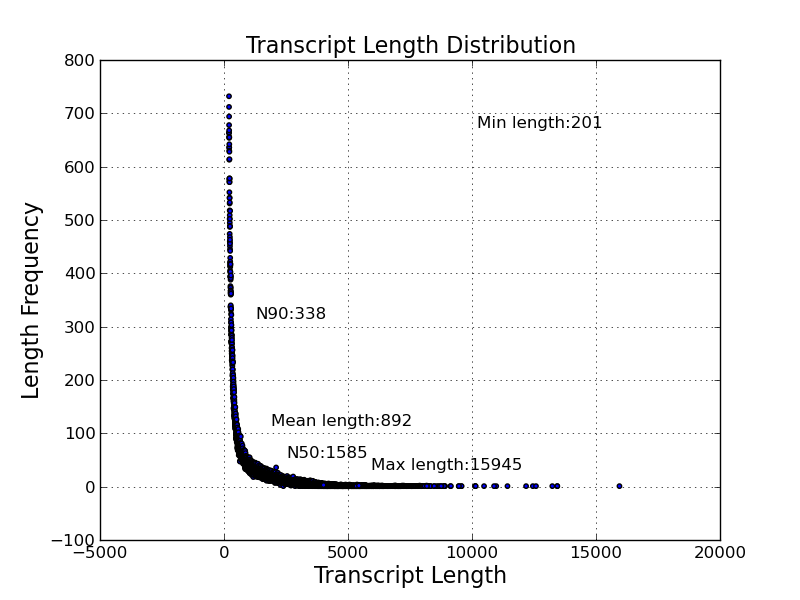

Supplement: S2 Fig — (TIF) [file pone.0175391.s013.tif]

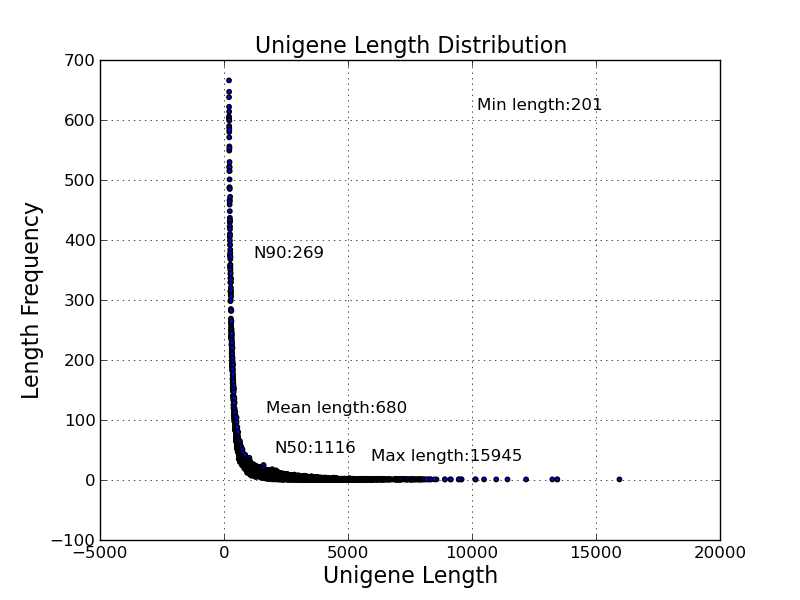

Supplement: S3 Fig — (TIF) [file pone.0175391.s014.tif]

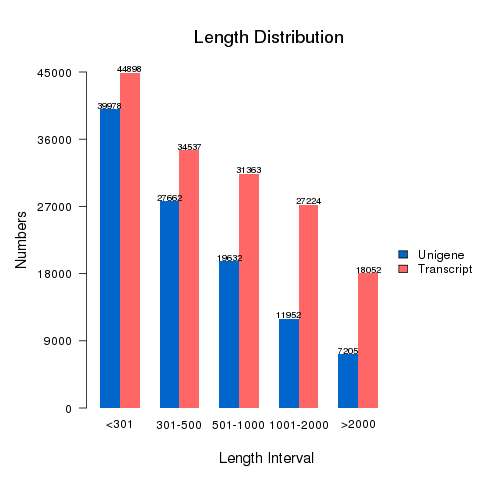

Supplement: S4 Fig — (TIF) [file pone.0175391.s015.tif]

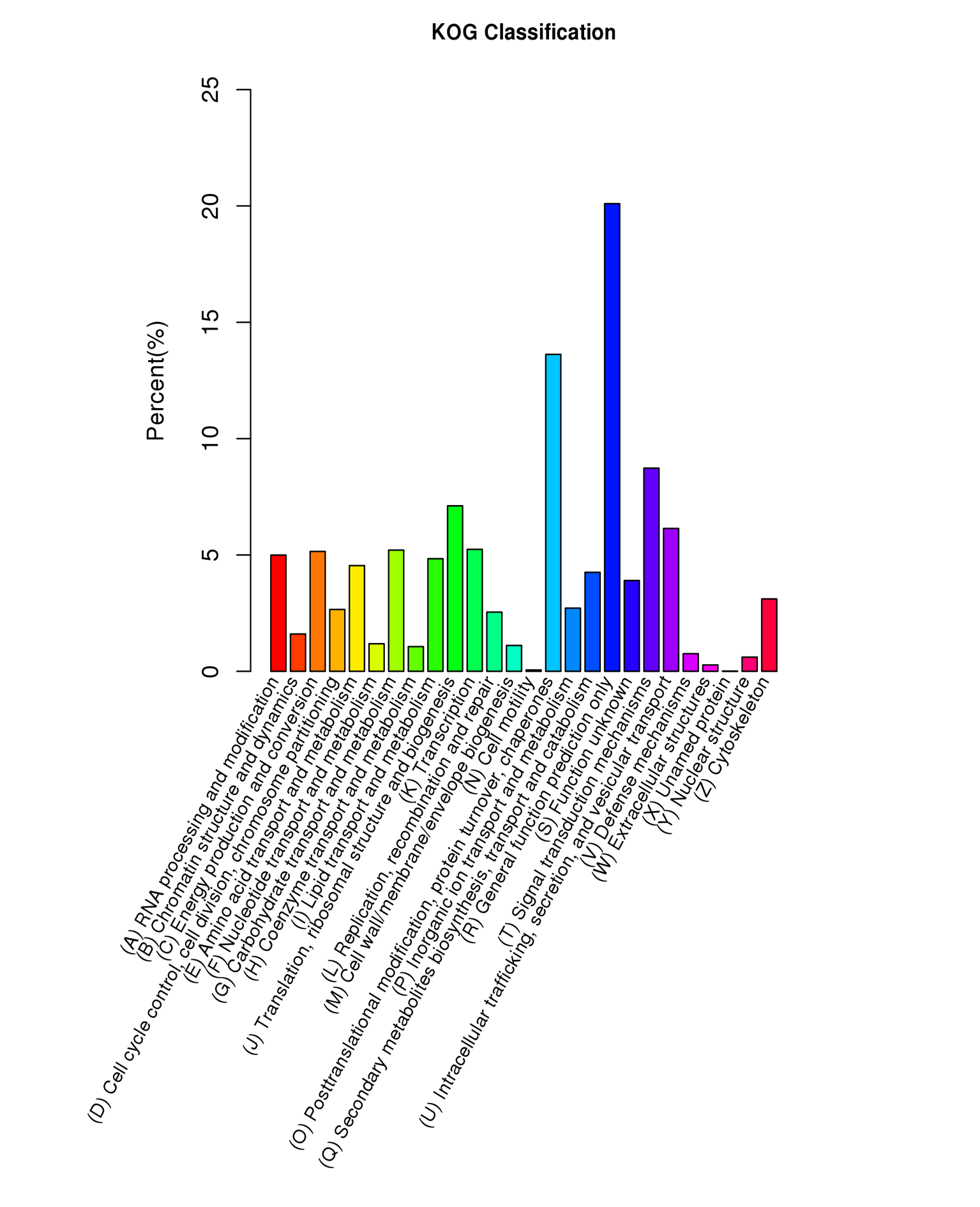

Supplement: S5 Fig — Unigenes aligned to the KOG database were classified into 26 functional classes. (TIF) [file pone.0175391.s016.tif]

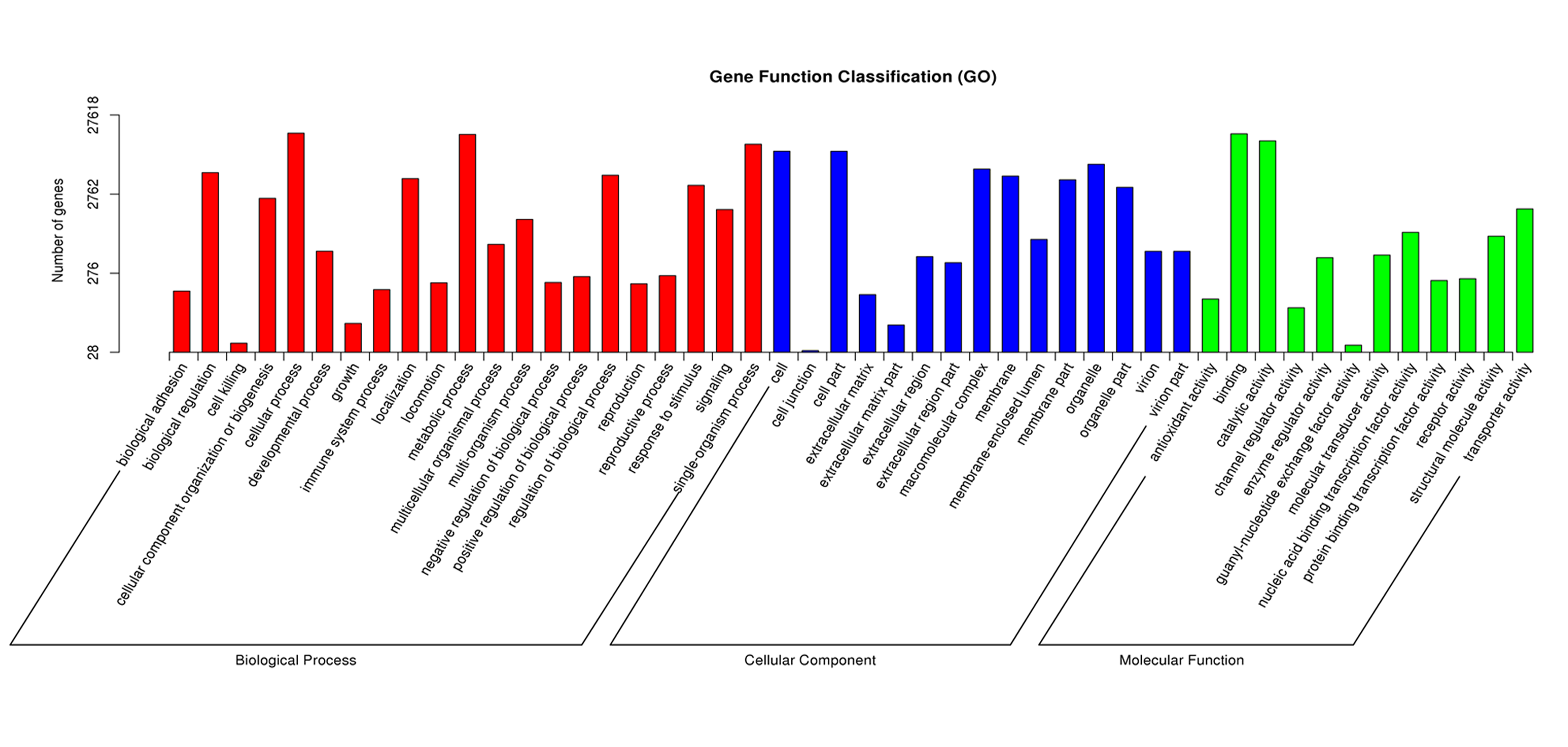

Supplement: S6 Fig — Unigenes with GO annotations were classified into three major functional categories (biological process, cellular components, and molecular functions). (TIF) [file pone.0175391.s017.tif]

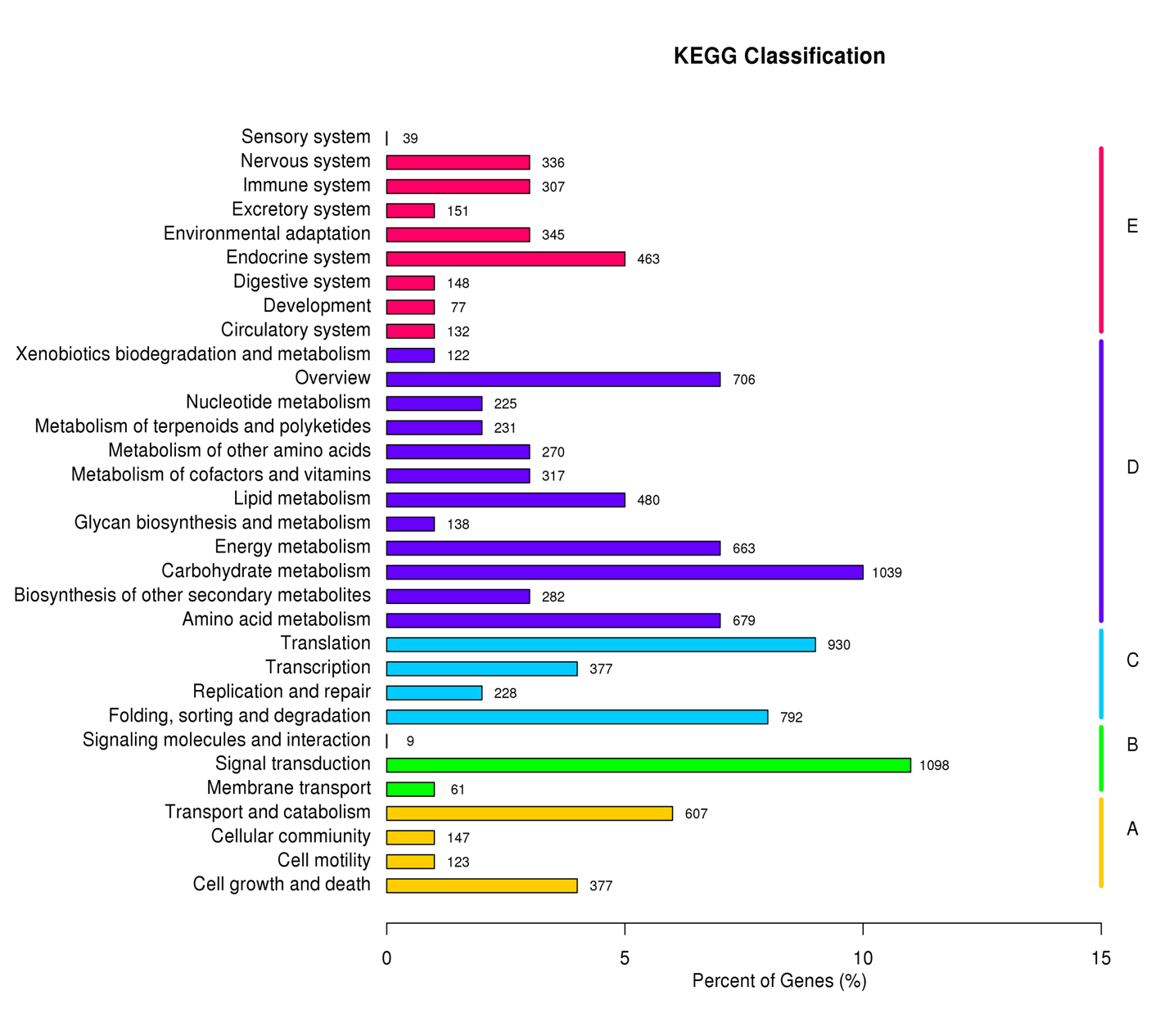

Supplement: S7 Fig — Unigenes with KEGG annotations were distributed in 272 KEGG pathways. (TIF) [file pone.0175391.s018.tif]

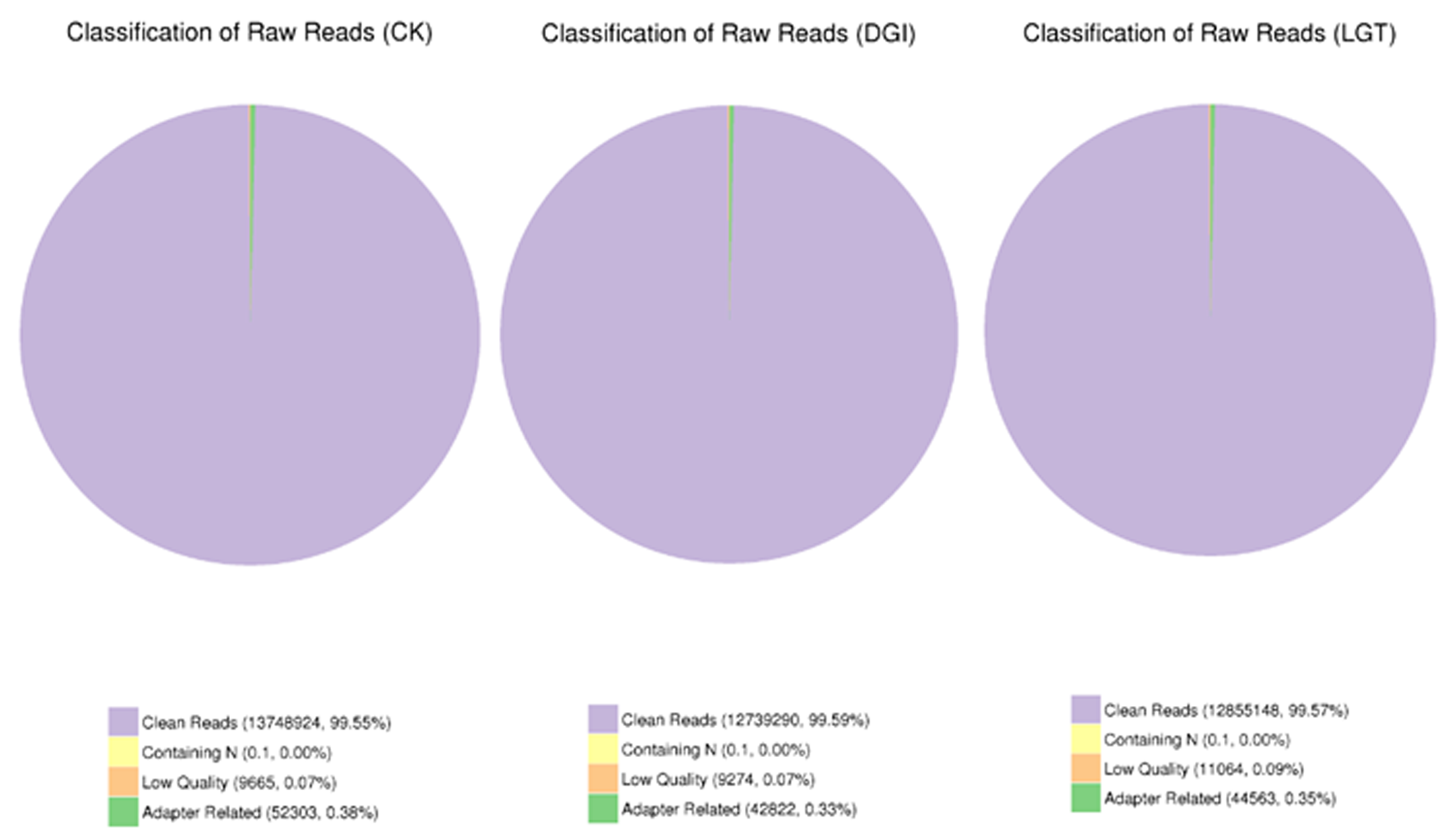

Supplement: S8 Fig — (TIF) [file pone.0175391.s019.tif]

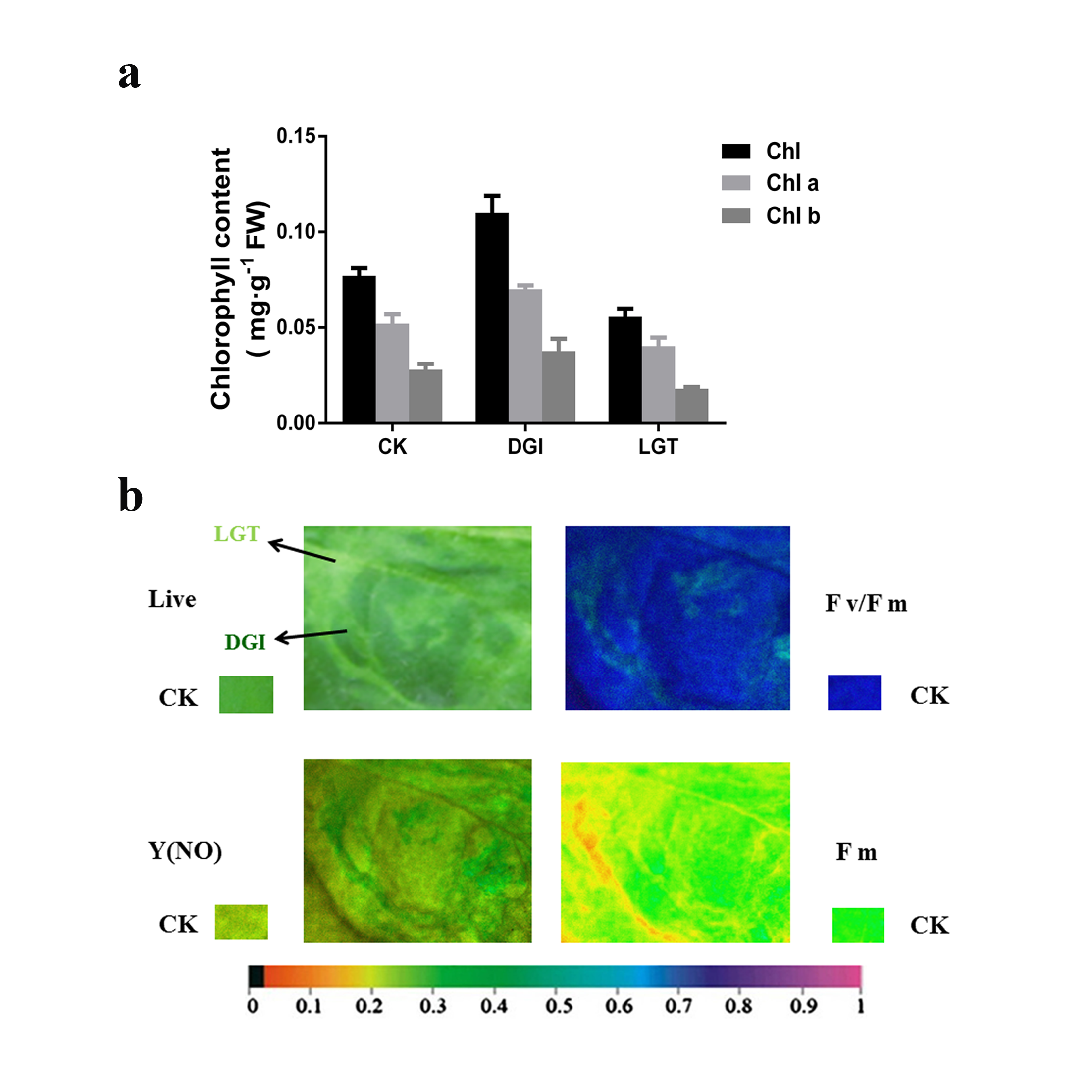

Supplement: S9 Fig — The error bars represent the standard deviations of the mean values that were obtained from three biological replicates (n = 3). (TIF) [file pone.0175391.s020.tif]

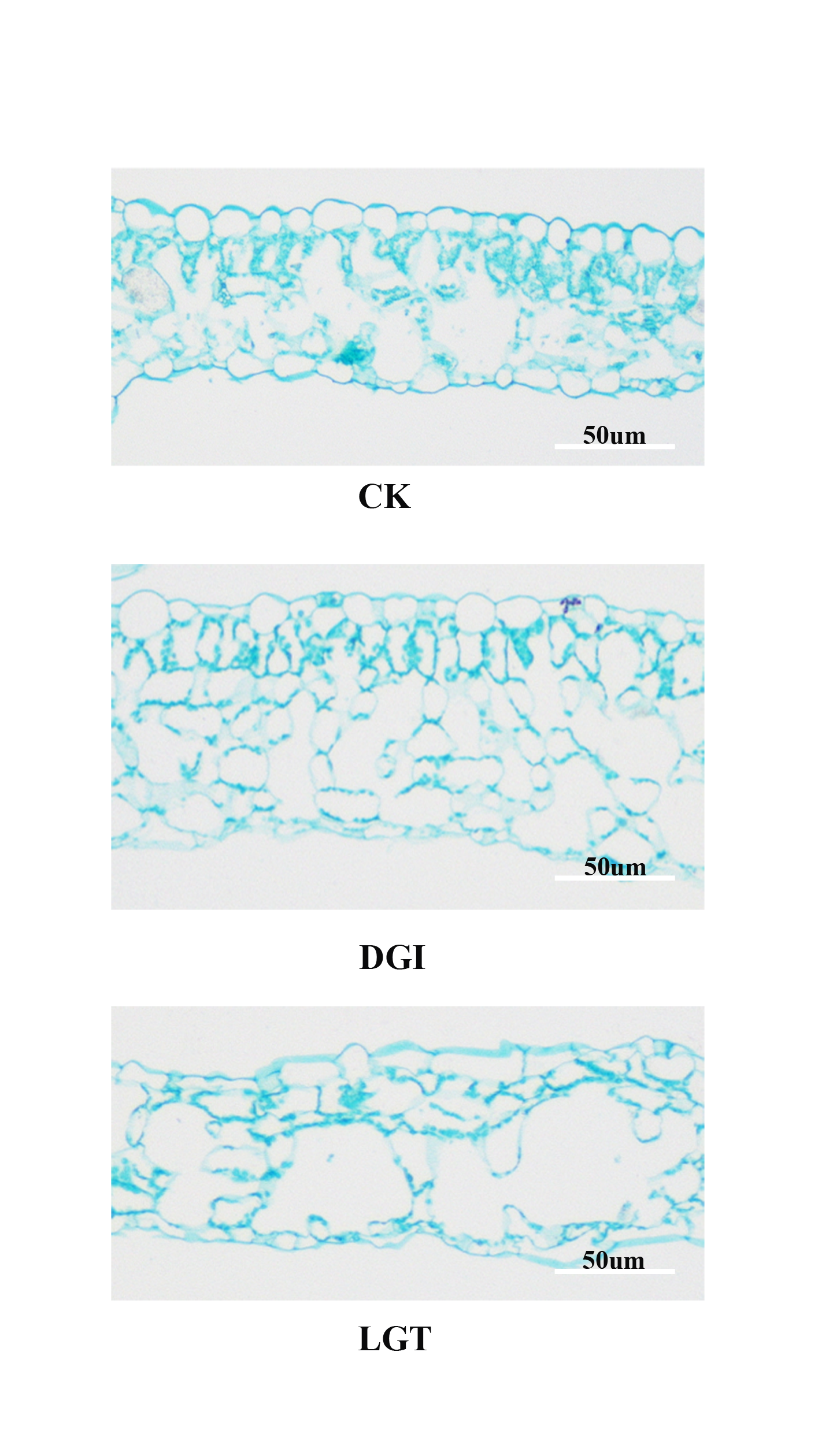

Supplement: S10 Fig — Scale bar = 50um. The experiments were repeated three times with similar results. (TIF) [file pone.0175391.s021.tif]

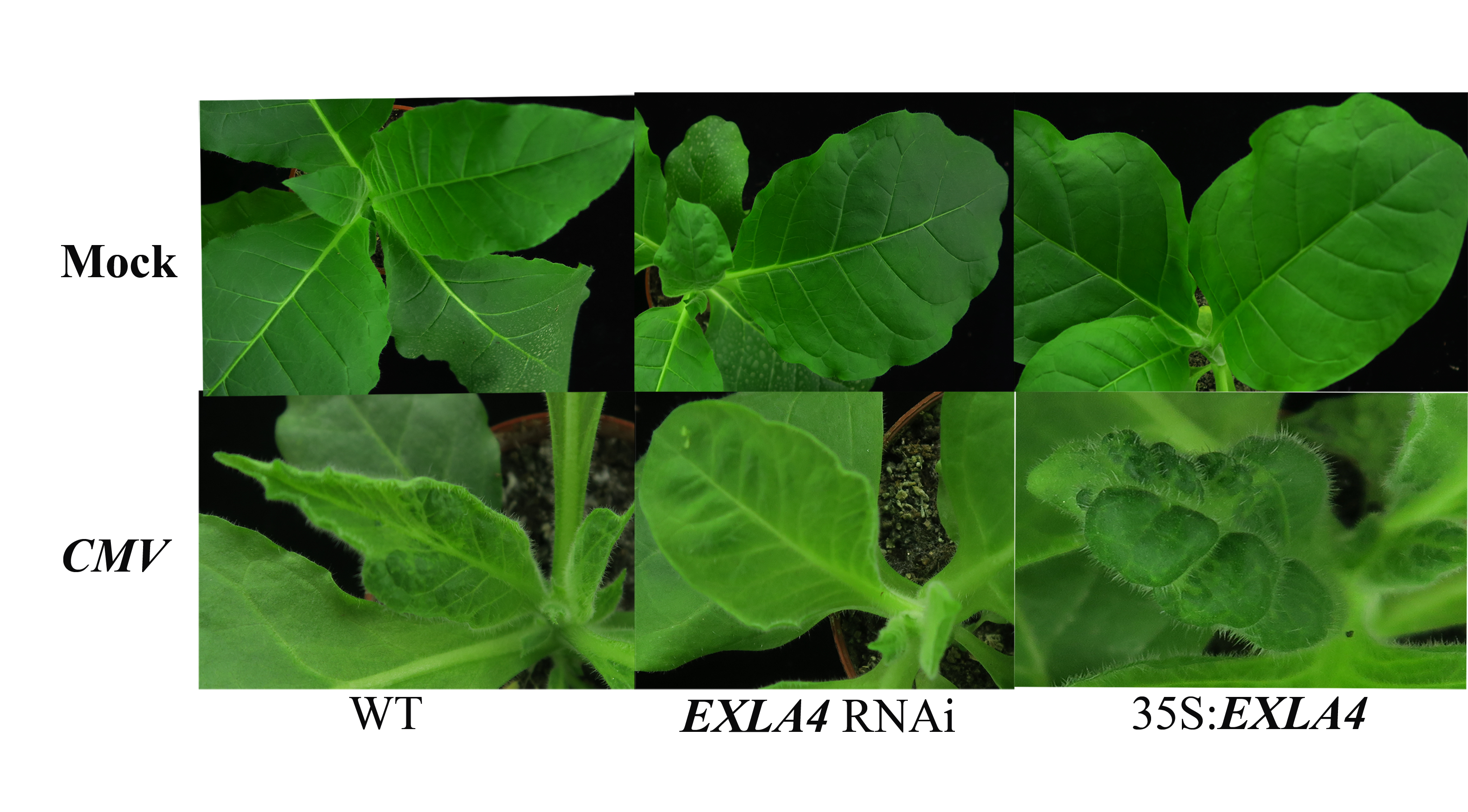

Supplement: S13 Fig — Mock, N. tabacum were mock inoculated with phosphate buffer; EXLA4-defective mutants (EXLA4 RNAi); overexpression lines (35S:EXLA4) and wild-type plants (WT). (TIF) [file pone.0175391.s024.tif]
