# Supplementary material for: RNA-seq approach to analysis of gene expression profiles in dark green islands and light green tissues of Cucumber mosaic virus-infected Nicotiana tabacum
Source: PLoS One. 2017 May 10;12(5):e0175391. doi: 10.1371/journal.pone.0175391 (PMC5425015; doi:10.1371/journal.pone.0175391)
Supplement: S1 Table — (DOCX) [file pone.0175391.s025.docx]

**Supplementary Table 1.** *Nicotiana tabacum* (*Nt*) gene primers be used in this study

| Gene | F primer | R primer |
| --- | --- | --- |
| H3.2 (c47264_g1) | GCACCAAGGAAGCAGTTAG | ACCCTCTTAGCGTGAATGG |
| EXPA1 (c46150_g2) | ATGGTGGAGGAGGTTGGAT | AGGTTGAAGTATGCGTGCC |
| WRKY33 (c46074_g1) | CATTCCCTAACTGTCCTA | TTCATTGTCACCCTTCCA |
| WRKY53 (c45397_g1) | TCCTTTGGCTTCTCCTGAA | CCTTGGATGAATAGACCCT |
| PR-4A (c35150_g1) | GTTGTGCGTGGCATTGTT | AGGTGGCCTTGCTGATAG |
| RPK1 (c51820_g1) | TATCCCTCTGTCTATCTC | TATTGGTCCATCAAACTC |
| RPK1(c52652_g1） | TAAACTCATCCTCGTCAC | GATACTAAACAAACTCCC |
| ERF4 (c39012_g1) | GAATCAGAGCCCAAGTCA | CCATAGGTGGAGCAAGGT |
| POR (c45425_g1) | ATGATTGATGGAGGAGAA | CGAGGAGGAGGTGTTGTT |
| POR (c38677_g1) | CCAATGGAGCAGTAAGGG | CAAGAGGACGACCAGACC |
| POR (c37871_g1) | CACGAAGAAACTGGCATTA | GACTTCCCATACTTTACGC |
| Cab (c41377_g1) | AGGCTGAACTGGTGAACG | GTTGCCGAGTGTTTGGAC |
