# Supplementary material for: RNA-seq approach to analysis of gene expression profiles in dark green islands and light green tissues of Cucumber mosaic virus-infected Nicotiana tabacum
Source: PLoS One. 2017 May 10;12(5):e0175391. doi: 10.1371/journal.pone.0175391 (PMC5425015; doi:10.1371/journal.pone.0175391)
Supplement: S2 Table — (DOCX) [file pone.0175391.s026.docx]

**Table S2.** Differentially expressed (log_2_Fold_change) of important genes in DGI vs CK, LGT vs CK and LGT vs DGI

| Gene ID | Swissprot Description | log_2_Fold_change (DGI vs CK) | | log_2_ Fold_change (LGT vs CK) log_2_ Fold_change (LGT vs DGI) | | |  |
| --- | --- | --- | --- | --- | --- | --- | --- |
| c34404_g1 | *Cucumber mosaic virus* movement protein | | 9.4565 | | 12.004 2.5264 |  | |
| c53149_g1  c77776_g1  c32491_g1  c33932_g1  c33932_g2  c39795_g1  c46150_g1  c46150_g2  c30809_g1  c32670_g1  c32670_g2  c37187_g1  c42002_g1  c42002_g2  c52671_g1  c39896_g1  c40192_g1  c46666_g1  c30481_g1  c42718_g1  c49565_g1 | *Cucumber mosaic virus* RNA-directed RNA polymerase 2a  *Cucumber mosaic virus* replication protein 1a  Auxin-binding protein ABP19a  Auxin-binding protein ABP19a  Auxin-binding protein ABP19a  Auxin-binding protein ABP19a  Expansin-A4  Expansin-A1  Aquaporin PIP2-7  Aquaporin PIP2-7  Aquaporin PIP2-7  Aquaporin PIP2-7  Aquaporin TIP2-1  Aquaporin TIP1-1  Endoribonuclease Dicer homolog 2    Superoxide dismutase  Peroxiredoxin Q  L-ascorbate oxidase homolog  Oxygen-evolving enhancer protein 2-3  Oxygen-evolving enhancer protein 1  Monocopper oxidase-like protein SKU5 | | 7.6939  8.0687  0.81243  2.2125  2.1766  2.1666  1.6274  1.5698  0.77125  1.4055  0.76324  0.81537  2.4755  2.7348  1.9758  0.73926  1.3028  1.0425  1.0656  0.91717  1.4779 | | 11.184 3.4688  11.412 3.3217    -1.9404 -2.774  -0.067123 -2.3008  -0.31417 -2.512  -0.24164 -2.4295    -0.61633 -1.0323  -0.10508 -1.6961      -0.61058 -1.403  -1.6774 -3.1041  -0.63121 -1.4156  -0.70235 -1.5389  0.7209 -1.7758  1.2911 -1.465  1.1534 -0.8436    -0.4425 -1.2029  -0.20995 -1.534  -0.50316 -1.5668  0.011672 -1.0751  -0.13792 -1.0763  -0.44293 -1.942 |  | |
